# Overexpression of *BoNAC019*, a NAC transcription factor from *Brassica oleracea*, negatively regulates the dehydration response and anthocyanin biosynthesis in *Arabidopsis*

**DOI:** 10.1038/s41598-018-31690-1

**Published:** 2018-09-06

**Authors:** Jinfang Wang, Weiran Lian, Yunyun Cao, Xiaoyun Wang, Gongle Wang, Chuandong Qi, Lun Liu, Sijia Qin, Xiaowei Yuan, Xingsheng Li, Shuxin Ren, Yang-Dong Guo

**Affiliations:** 1 College of Horticulture, Beijing Key Laboratory of Growth and Developmental Regulation for Protected Vegetable Crops, China Agricultural University, Beijing, 100193 China; 2 Shandong Huasheng Agriculture Co., Ltd, Shandong, China; 3 School of Agriculture, Virginia State University, PO Box 9061, Petersburg, VA 23806 USA

## Abstract

NACs are one of the largest transcription factor families in plants and are involved in the response to abiotic stress. *BoNAC019*, a homologue of *AtNAC019*, was isolated from cabbage (*Brassica oleracea*). BoNAC019 was localized in the nucleus and functioned as a transcriptional activator. The expression of *BoNAC019* was induced by dehydration, salt, abscisic acid (ABA), and H_2_O_2_ treatments. *BoNAC019* overexpressing plants were generated to explore the function of *BoNAC019* in response to drought stress. Overexpression (OE) of *BoNAC019* reduced drought tolerance with lower survival rate, higher water loss rate, lower proline content and ABA content. The seed germination and root length assays of *BoNAC019*-OE plants showed decreased sensitivity to ABA. Under drought condition, antioxidant enzymes and anthocyanin content decreased in *BoNAC019* -OE plants, resulting in the accumulation of more reactive oxygen species (ROS), which cause damage to plants. Several stress-responsive genes, antioxidant enzymatic genes, anthocyanin biosynthetic genes and ABA signaling genes were down-regulated under drought condition while the ABA catabolism genes were induced in *BoNAC019*-OE plants under both normal and drought conditions. Our results demonstrated that *BoNAC019* might participated in regulating drought tolerance by inducing ABA catabolism genes and decreasing ABA content.

## Introduction

Drought stress induces lots of changes in plants and limits plant growth, development, and productivity^1–3^. A set of strategies were evolved to cope with drought stress in plants, including shortening the life cycle, reducing water loss, adjusting osmotic content, and altering gene expression and cellular metabolism^4–6^. Transcription factors play important roles in different biological processes^7–9^.

NACs are one of the largest transcription factor families in plants, it has been reported in many species. There are 117 NAC genes in *Arabidopsis*, 151 in rice, and 152 each in soybean and tobacco^10–12^. The C-terminal region of NAC proteins is transcriptional region, and the N-terminal region is highly conserved, and can be divided into five subdomains (A–E)^13–15^.

NAC transcription factors play key roles in complex drought signaling processes^16,17^. *AtNAC019*, *AtNAC055*, and *AtNAC072* were stress-responsive NAC genes, the expressions of these genes were induced by drought treatment. Overexpressing *AtNAC019*, *AtNAC055*, and *AtNAC072* improved drought tolerance and up-regulated the expression of *ERD1* (*early responsive to drought 1*)^16,18^. *OsNAC1* was reported as a stress responsive NAC gene, overexpressing *OsNAC1* enhanced drought tolerance in transgenic rice, and lots of stress-responsive genes were induced in *OsNAC1* overexpressing plants. *OsERD1* was verified as the target gene of *OsNAC1*^17^. Overexpressing *OsNAC3* in rice showed improved tolerance to heat and drought stresses in transgenic plants. Moreover, *OsNAC3* directly regulated the expression of five ROS-associated genes^19^. *RhNAC3* was reported as a novel rose NAC and induced by dehydration. Overexpressing *RhNAC3* improved drought tolerance in transgenic *Arabidopsis*, and many genes respond to stress were induced in overexpressing lines^20^.

Abiotic acid (ABA) is the most important phytohormone for plants to resist abiotic stresses, especially for drought stress. When plants suffered from drought stress, ABA content is significantly increased and might result in complex changes, such as stomatal closure, inducing the expression of numerous stress responsive genes and eventually leading to physiological responses^21,22^. In last twenty years, ABA synthesis and signaling genes has been widely studied in different species. The synthesis gene NCED (9-cis-epoxycarotenoid dioxygenase) was cloned from crops and shown to help plants to resist drought stress^23,24^. For ABA signaling, the receptors named PYRs (pyrabactin resistances) were reported in 2009. Overexpressing these genes have been verified to improve drought stress resistance in *Arabidopsis*^25,26^. Overexpression of *CsATAF1* enhanced the hypersensitivity to ABA and drought tolerance by directly regulating the expressions of *CsDREB2C* and *CsABI5*^27^.

Adverse environmental conditions lead to the accumulation of ROS in plants^28,29^. Enzymatic antioxidants and non-enzymatic antioxidants, including ascorbate, glutathione (GSH), carotenoids, tocopherols, and flavonoids are defense systems for scavenging ROS in plants^30,31^.

Anthocyanins are water soluble flavonoid pigments in plants. A variety of stress factors affect anthocyanin biosynthesis and accumulation in plants^32,33^. Drought conditions promote anthocyanin synthesis to improve drought resistance by scavenging ROS^34,35^. Chalcone synthase (CHS), flavanone 3-hydroxylase (F3H), flavonoid 3′-hydroxylase (F3′H), Phenylalanine ammonia-lyase (PAL), cinnamic, leucoanthocyanidin dioxygenase (LDOX), acid 4-hydroxylase (C4H), dihydroflavonol 4-reductase (DFR), chalcone isomerase (CHI), and UDP -glucose: flavonoid 3-O-glucosyltransferase (UFGT) are important enzymes in the anthocyanin biosynthetic pathway. The MYB (TT2, PAP1, PAP2, MYB113, and MYB114) and bHLH (TT8, GL3, and EGL3) transcription factors interacted with the WD40 protein (TTG1) to regulate these anthocyanin biosynthetic genes^36–40^. Several NAC genes have been identified to participate in anthocyanin biosynthesis. Under a high light stress condition, *AtNAC078* positively regulated anthocyanin production^41^, while *JUB1/ANAC042* and *AtNAC032* negatively regulated anthocyanin biosynthesis^33,42^.

*Brassica oleracea* is one of the most important vegetables of the *Brassica* species, and there are 271 NAC genes in the Chinese cabbage genome (http://brassicadb.org/brad/index.php)^43,44^. However, the functions of NAC transcription factors in response to abiotic stress and anthocyanin biosynthesis have not been reported in cabbage.

The expression of *BoNAC019* was induced by abiotic stress treatments. *BoNAC019*-OE reduced drought tolerance in *Arabidopsis*, with higher water loss rates and higher MDA and proline contents. Overexpression of *BoNAC019* accumulated more ROS and decreased antioxidant enzyme activities. QPCR experiments showed that the expressions of many stress responsive genes decreased in the OE lines.

Overexpression of *BoNAC019* also reduced anthocyanin accumulation under drought conditions. Compared with the WT plants, anthocyanin content was much lower in *BoNAC019*-OE plants, and the expressions of anthocyanin biosynthetic genes decreased in *BoNAC019*-OE plants. These results showed that *BoNAC019* negatively regulated the tolerance to drought stress and anthocyanin biosynthesis.

## Methods and Materials

### Cloning and sequence analysis of BoNAC019

According to the *BoNAC019* cDNA sequence, the BoNAC019 gene was cloned and the primers were showed in supporting information Table S1. DNAman software was used to analyze the homology between BoNAC019 and the NAC proteins of other species, and the Neighbor-Joining (NJ) algorithm in MEGA program (ver. 5.0) was used to construct the phylogenetic tree.

### Subcellular localization of the BoNAC019 protein

The pCAMBIA 1302 vector (Addgene, Cambridge, MA, USA) was used to analyze the subcellular location of BoNAC019. The fusion constructs (*BoNAC019*-GFP) and empty vector (GFP) were transformed by particle bombardment. The confocal microscopy (Nikon Inc., Melville, NY, USA) was used to observe these epidermal cells of onion with and without fluorescence after a 26 h incubation in the dark at 26 °C.

### Transactivation assay of BoNAC019

The PCR products of *BoNAC019* (GAL4BD - *BoNAC019*-FL^1–361^), the N-terminus of *BoNAC019* (GAL4-BD- *BoNAC019*-N^1–171^) and the C-terminus of *BoNAC019* (GAL4-BD- *BoNAC019*-C^171–361^) were fused into the GAL4-BD vector (Table S1). The vectors were transferred into *Arabidopsis* protoplasts^20^. The luciferase activity was measured by luminometer.

### Growth conditions for the plant materials and qPCR analysis

The cabbage line studied was ‘Zhonggan-11’. The cabbage seedlings were planted under a 16 h light/8 h dark cycle were subjected to different stress treatments. Four-week-old cabbage seedlings were transferred into Hoagland nutrient solution containing 150 mM NaCl, 10% PEG, 10% H_2_O_2_, or 100 μM ABA for the indicated times. Leaves were collected at the designated time points after different stress treatments.

The qPCR was performed according to our laboratory’s own method described previously^27^. Three biological replicates were performed for each sample. The primer sequences utilized are listed in Supplemental Table 1.

### Plant transformation and evaluation of drought stress tolerance

To investigate the function of *BoNAC019*, overexpressing *Arabidopsis* plants were generated. The *BoNAC019* cDNA was fused into pBI121 (Table S1). The *Agrobacterium tumefaciens* strain GV3101 contained these constructs were transformed them into *Arabidopsis* Col-0 plants using the floral dip method^45^. Transgenic plant seeds were selected in MS containing kanamycin (80 mg/L). The positive plants were screened by PCR and qPCR.

Before the drought treatment, forty T_3_ transgenic plants and WT plants were grown for four weeks at 22 °C/16 °C and under a 16 h/8 h light/dark cycle. For drought assay, these seedlings were treated without watering for three weeks. The mock control (CK) were the seedlings treated with water. These drought treatments experiments were repeated three times.

### Physiological index of transgenic plants and WT plants

The water loss rate and MDA content in leaves were measured according to Mao *et al*.^46^ and Zhang *et al*.^47^. Proline content and ABA content were measured according to Szekely *et al*.^48^ and Wang *et al*.^49^. H_2_O_2_ content and stained with DAB, superoxide dismutase (SOD), peroxidase (POD), and catalase (CAT) activities were measured in leaves using a method described previously^19,50–52^.

### Seed germination and root length of transgenic and WT plants

Approximately 100 seeds of WT plants and *BoNAC019*-OE plants were germinated on MS medium containing 1 μM ABA for one week. The germination rate was calculated based on radicle protrusion. Each experiment was performed in triplicate. Seedlings grown on MS medium for five days were transferred to MS medium containing 1 μM ABA for five days. Each experiment was performed in triplicate. The mock control (CK) were the seedlings without ABA treatment.

### Stomatal closure and aperture

We used epidermal strips of *Arabidopsis* for measuring the stomatal aperture. We merged the epidermal strips into the 30 mM KCl and 10 mM MES-KOH (pH 6.15) solution for 2.5 h at 22 °C and put them under light for fully opening the stomata^53–55^. Then, added ABA to the same solution for 2.5 h more. We measured more than 130 stomata of each lines using IMAGEJ 1.36b software (Broken Symmetry Software). Each experiment was performed in triplicates.

### Anthocyanin content

WT plants and transgenic plants were germinated on MS medium for five days and were then transferred to MS containing 100 mM mannitol for five days to assess dehydration-induced accumulation of anthocyanin.

The absorbance spectra of anthocyanin are strikingly different with a change in pH^56^. The anthocyanin content of WT plants and *BoNAC019*-OE plants leaves were measured according to Zhang *et al*.^32^.

## Results

### Sequence analysis and transcriptional activation of the BoNAC019 gene

**Figure 1:**
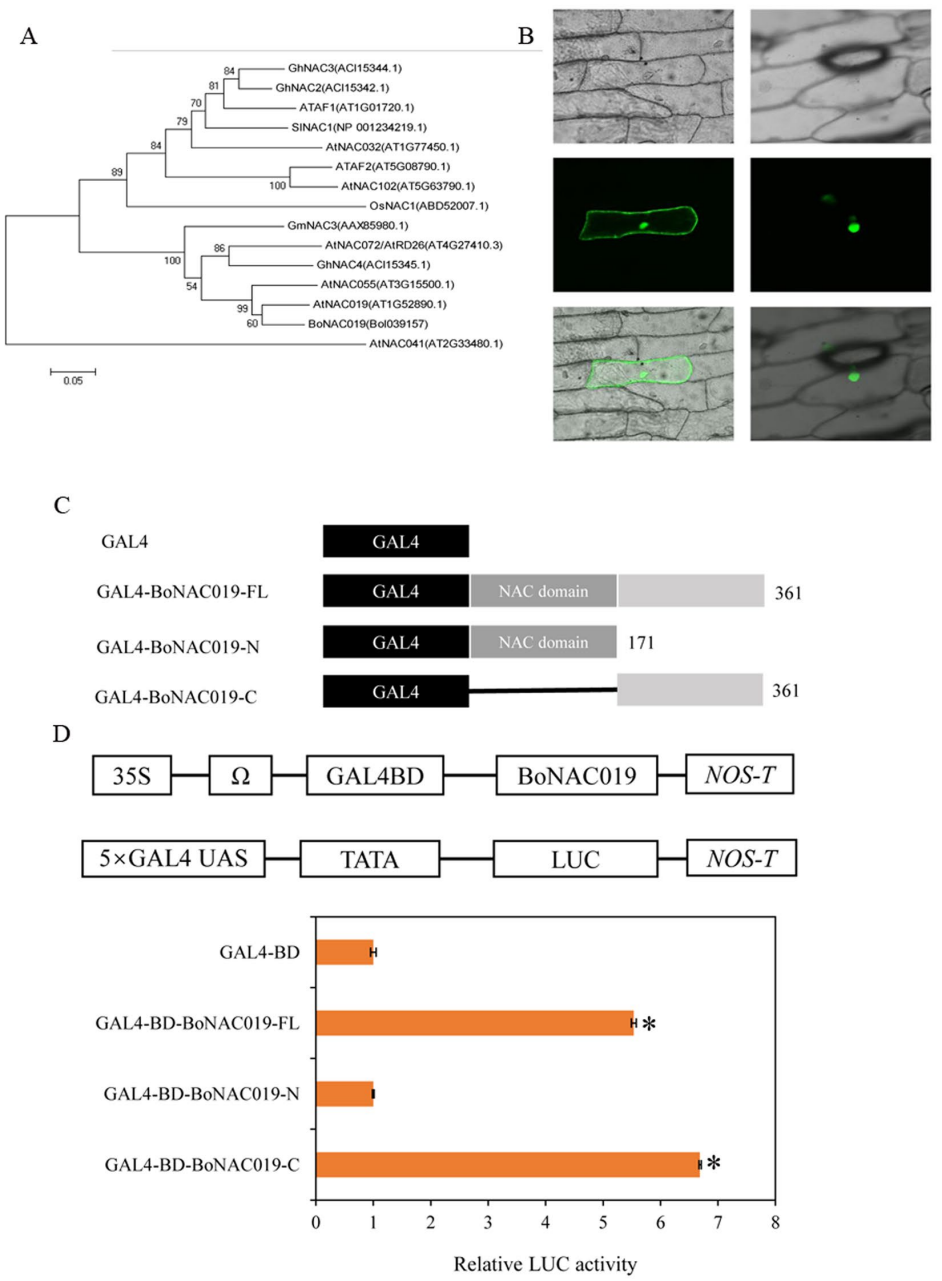
Sequence analysis of BoNAC019, nuclear localization and transcriptional activation of BoNAC019. (**A**) Phylogenetic tree of BoNAC019 and NAC members from other plant species. (**B**) Subcellular localization of the BoNAC019 protein in onion epidermal cells. (**C**) Transcription activation activity of BoNAC019. The full-length proteins (BoNAC019-FL), N-terminal fragment (BoNAC019-N) and C-terminal fragment (BoNAC019-C) were fused with the vector GAL4BD. (**D**) The plasmids containing the fusion genes and the empty control plasmid pGBKT7 were introduced into protoplasts. The GAL4BD vector was used as a negative control.

*BoNAC019* (Bol039157) was cloned from cabbage and was the closest homologue to *AtNAC019* gene (AT1G52890) in *Arabidopsis*. The multiple sequence alignment analysis showed that the N-terminal domain was highly conserved, while the C- terminal domain had low similarity with *Arabidopsis* proteins (Fig. S1). BoNAC019 clustered in the same clade as AtNAC019 and AtNAC055 by phylogenetic tree analysis (Fig. 1A). *BoNAC019* is a putative transcription factor, and transient expression assays showed that *BoNAC019* was located in nuclei (Fig. 1B).

A GAL4 transient expression assay was used to investigate the transcriptional activity of BoNAC019 in *Arabidopsis* protoplasts. The relative LUC activities of *Arabidopsis* protoplasts transformed with GAL4BD-*BoNAC019*, GAL4BD-BoNAC019-C were significantly higher than negative control and GAL4BD-BoNAC019-N (Fig. 1C,D). These results showed that BoNAC019 functioned as a transcriptional activator.

### Expression pattern of BoNAC019

**Figure 2:**
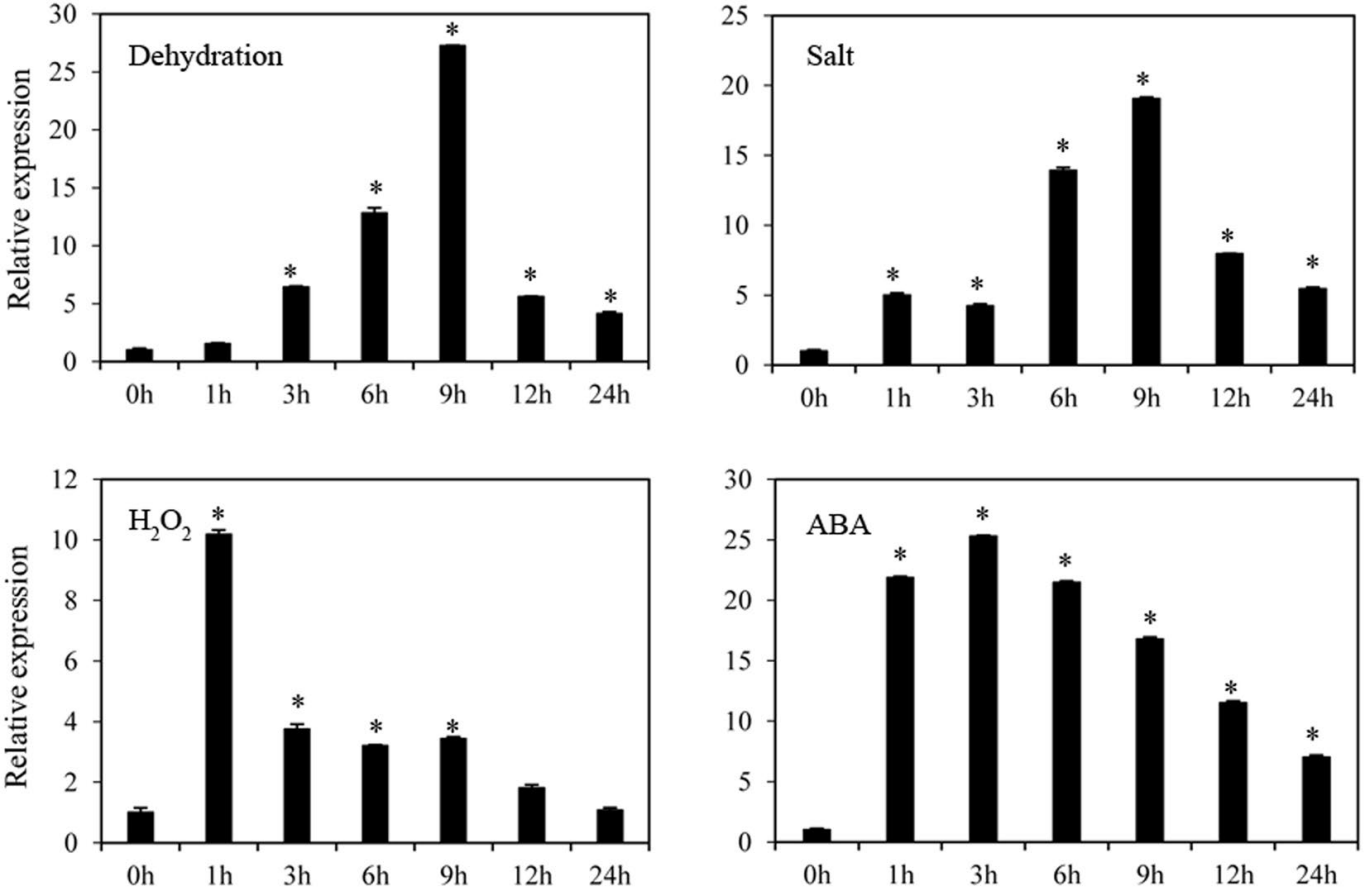
Expression pattern of *BoNAC019* in cabbage after stress treatments. Expression pattern of *BoNAC019* in cabbage leaves after 150 mmol/L NaCl, 10% PEG6000, 10% H_2_O_2_, and 100 μM ABA treatments was performed via qPCR analysis. *BoActin* was used as the endogenous control. Error bars show the standard deviations for three independent replicates. Asterisks indicate statistically significant differences from controls (*P < 0.05).

A variety of NAC transcription factors have been reported to respond to abiotic stress. QPCR experiments were used to detect the expression of *BoNAC019* to further test whether *BoNAC019* responds to abiotic stress. Under the dehydration treatment, *BoNAC019* expression increased more than 25-fold after 12 h. Under the salt treatment, *BoNAC019* expression increased more than 20-fold after 9 h. *BoNAC019* expression was increased more than 12- and 20-fold under the ABA and H_2_O_2_ treatments, respectively (Fig. 2).

### Overexpression of BoNAC019 reduces tolerance to drought in Arabidopsis

To investigate whether *BoNAC019* functions in the response to drought stress, transgenic *Arabidopsis* plants were generated. After kanamycin resistance and PCR analyses, four transgenic lines were selected for further study. Among these, the expression levels of *BoNAC019* in the OE1 and OE2 lines were much higher than those in the other lines by qPCR analysis (Fig. S2).

**Figure 3:**
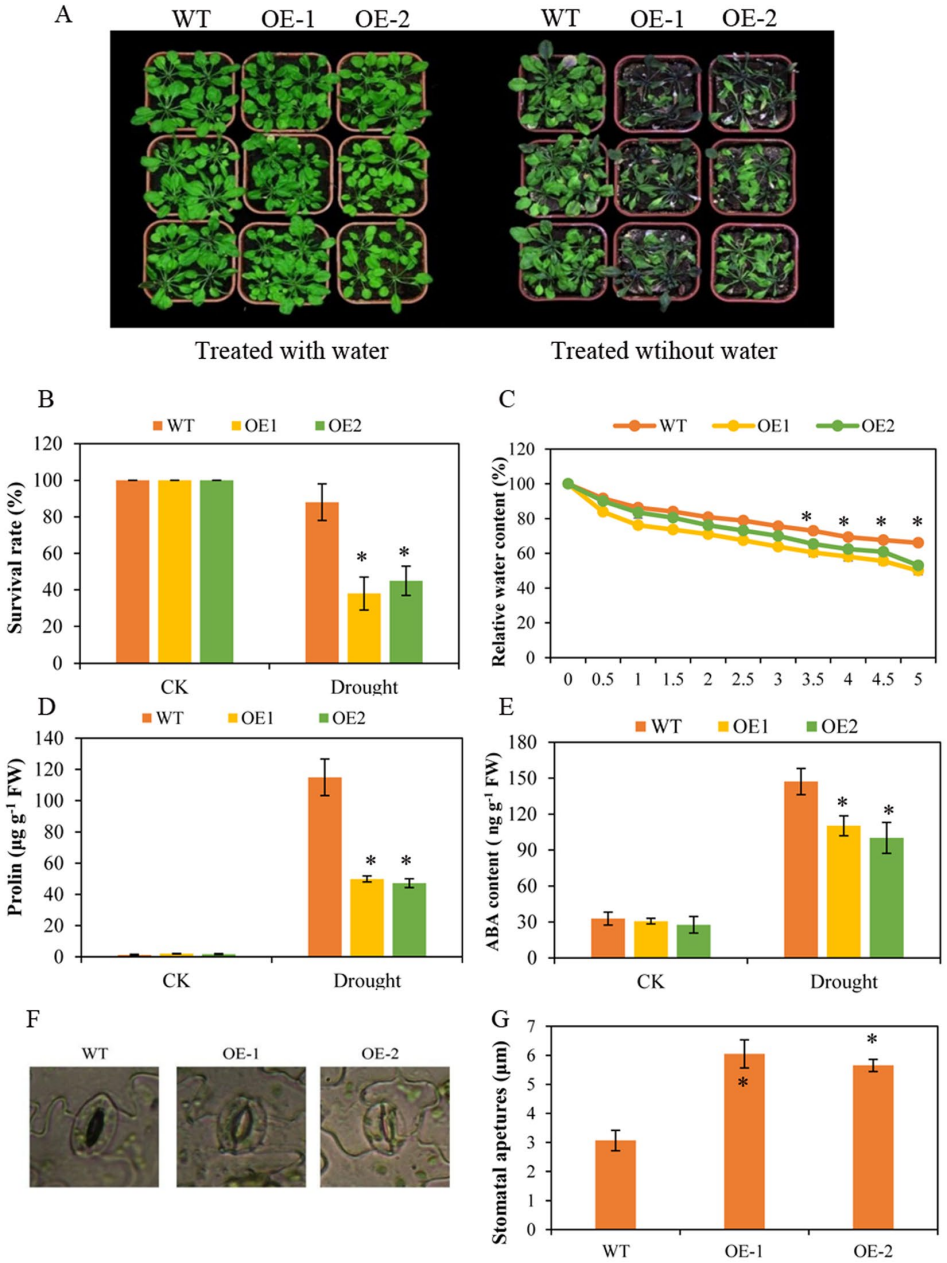
Overexpression of *BoNAC019* reduced tolerance to drought stress. (**A**) Phenotypes of WT and OE plants after water withholding for three weeks. (**B**) Survival rate of WT and OE plants after drought treatment. (**C**) Water loss rate of the detached leaves. (**D**) Proline content. (**E**) MDA content. (**F**,**G**) Representative images (**F**) and stomatal aperture (**G**) of WT and OE plants after drought treatment. Seedlings treated with water were used as a mock control (CK). Error bars show the standard deviations for three independent replicates. Asterisks indicate statistically significant differences compared to WT at the same time point (*P < 0.05).

The growth performances of WT plants and transgenic plants were basically similar with well-watering condition. Without watering for three weeks, only 38% of the *BoNAC019*-OE plants survived, and was significantly lower than that of WT plants. Leaf wilting of *BoNAC019*-OE plants was much more serious than that observed in WT plants (Fig. 3A,B). Relative water content (RWC) in the OE1 and OE2 lines decreased to 50% and 53%, respectively after a 5 h incubation, while WT plants retained almost 65% of their fresh weight (Fig. 3C), and the stomatal apertures of overexpressing plants were larger than those of WT plants (Fig. 3F,G). These results indicating that the water retaining capacity of overexpressing plants was weaker. The free proline content and ABA content increased under abiotic stress conditions to cope with these stresses, the results showed that after drought treatment, the proline and ABA contents of WT plants and OE plants increased and proline and ABA contents were much lower in OE plants (Fig. 3D,E). *BoNAC019* as the closest homologue gene to *AtNAC019*, the growth performances of the overexpression plants in response to drought stress were quite different. Overexpression of *AtNAC019* showed higher survival rate and more tolerant to drought (Fig. S3).

### Overexpression of BoNAC019 decreases sensitivity to ABA

**Figure 4:**
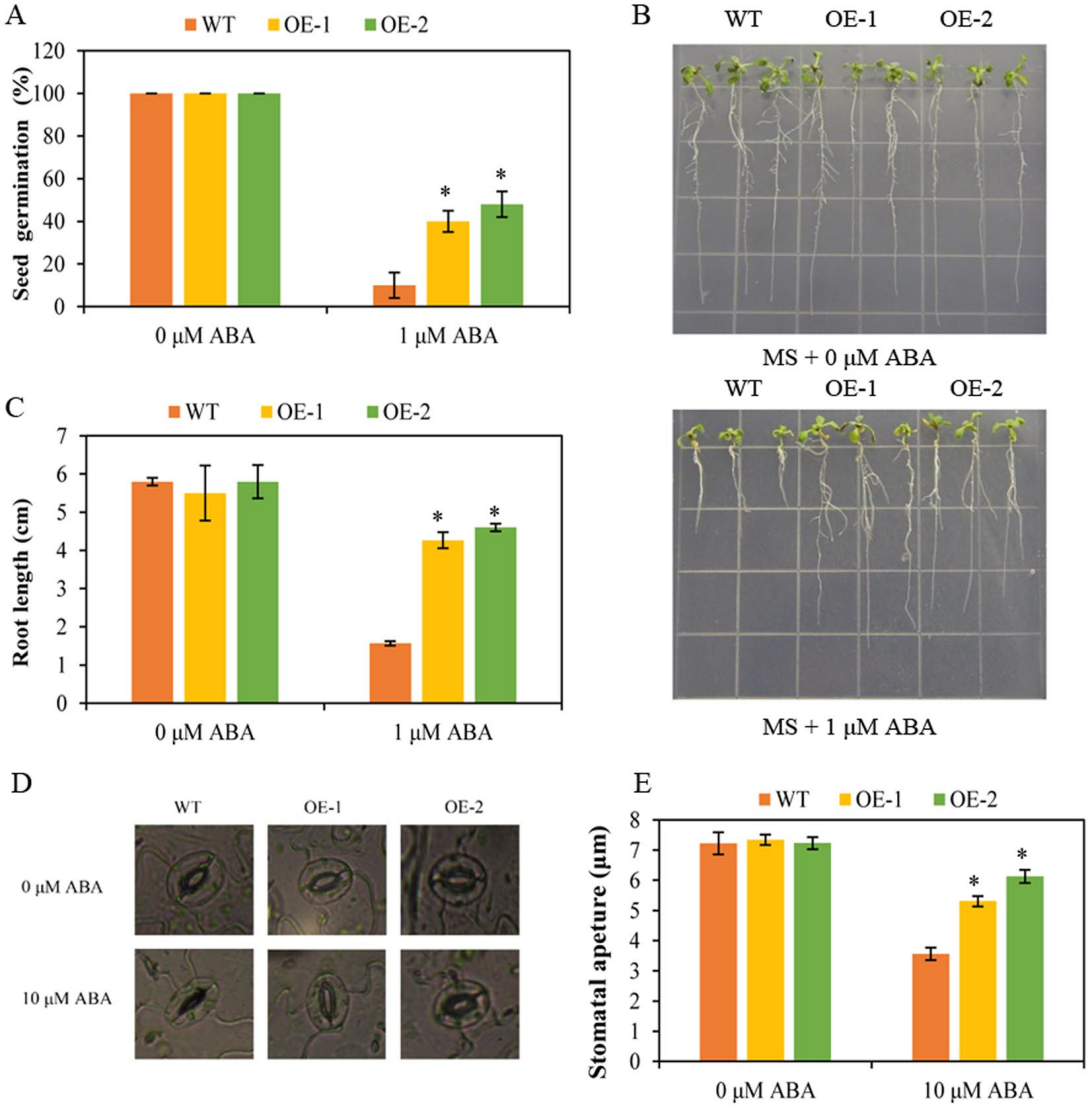
Overexpression of *BoNAC019* increased sensitivity to ABA. (**A**) Seed germination rate of WT and OE plants under ABA treatment. (**B**) Comparison of primary root length of WT and OE plants with ABA treatment. Five-day old seedlings grown on MS medium containing 1 μM ABA for 5 days. (**C**) Quantification of primary root length. (**D**,**E**) Representative images (**D**) and stomatal aperture (**E**) of WT and OE plants before and after ABA treatments. Seedlings treated without ABA were used as a mock control (CK). Error bars show the standard deviations for three independent replicates. Asterisks indicate statistically significant differences compared to WT at the same time point (*P < 0.05).

ABA is an important hormone involved in drought stress resistance in plants^57,58^. The germination rate and root elongation of *BoNAC019*-OE plants were analyzed to understand the role of *BoNAC019* in the ABA signaling pathway. No obvious difference was observed between the germination rate of WT and *BoNAC019* OE plants after 5 days of germination on MS medium without ABA. However, the germination rate of *BoNAC019* OE plants was much higher than that in WT plants when the MS medium contained 1 μM ABA (Fig. 4A). The root elongation analysis showed no obvious difference in root lengths between WT and *BoNAC019*-OE plants, but root lengths of *BoNAC019*-OE plants were longer than those of WT plants on MS medium containing 1 μM ABA (Fig. 4B,C). ABA also mediates stomatal closure^57,59^. The stomatal apertures of *BoNAC019*-OE plants decreased by 20% in the 2.5 h 10 μM ABA treatment, while WT plants decreased by 20% (Fig. 4D,E). These results show that overexpressing *BoNAC019* decreases sensitivity to ABA.

### Overexpression of BoNAC019 increases H_2_O_2_ and reduces antioxidant enzyme activities

**Figure 5:**
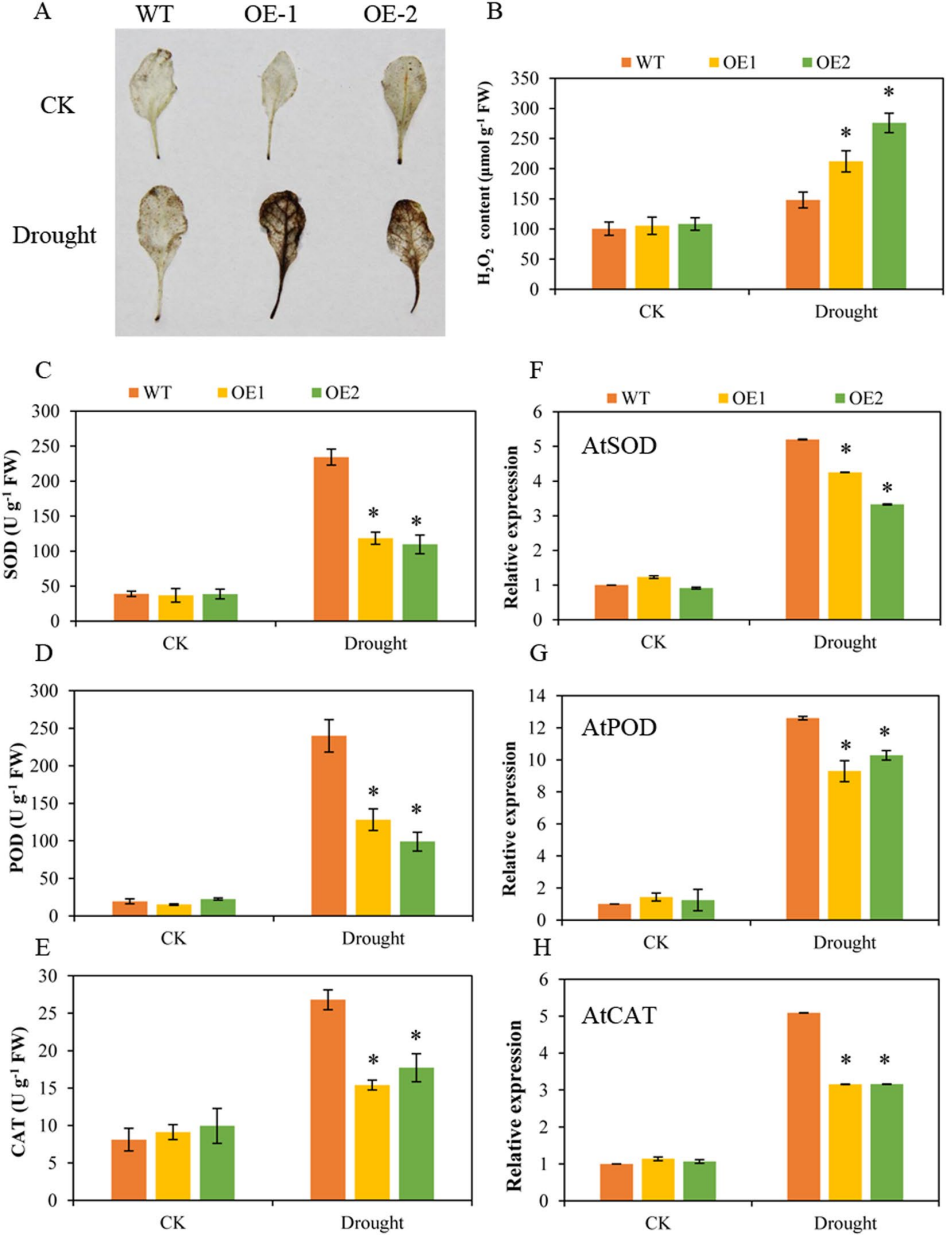
Overexpression of *BoNAC019* accumulated H_2_O_2_ and decreased antioxidant enzyme activity under drought condition. (**A**) Histochemical staining of WT and OE plants by diaminobenzidine (DAB) under normal and drought conditions. (**B**) H_2_O_2_ content of WT and OE plants under normal and drought conditions. (**C**–**E**) SOD, POD and CAT activity of WT and OE plants under normal and drought conditions. (**F**–**H**) The relative expressions of AtSOD, AtPOD and AtCAT. Seedlings treated without ABA were used as a mock control (CK). Error bars show the standard deviations for three independent replicates. Asterisks indicate statistically significant differences compared to WT at the same time point (*P < 0.05).

Adverse environmental conditions cause ROS accumulation in plants. Histochemical staining with diaminobenzidine (DAB) was used to detect the accumulation of ROS in WT plants and *BoNAC019*-OE plants. There was no obvious differences in leaf staining were observed between WT plants and *BoNAC019*-OE plants under normal condition, they stained negligibly with DAB. Under drought condition, the leaves of *BoNAC019*-OE plants were stained deeper than those of WT plants (Fig. 5A). The H_2_O_2_ content of *BoNAC019*-OE plants was much higher, which was consistent with the leaf staining result (Fig. 5B).

Enhanced antioxidant enzyme activities are an important way to scavenge ROS in plants. To further detect the ability of plants to scavenge ROS, the activities of the antioxidant enzymes SOD, POD, and CAT were evaluated. Under normal condition, no obvious differences in the activities of SOD, POD and CAT were detected in WT plants or *BoNAC019*-OE plant. Under drought treatment, the SOD, POD, and CAT activities were increased in both *BoNAC019*-OE plants and WT plants, but the enzyme activities were much lower in *BoNAC019*-OE plants than those in WT plants (Fig. 5C–E). These genes were reported encoding these antioxidant enzymes (AtSOD, AtPOD, and AtCAT) were selected for further study. As results, the expressions of these genes were much lower in *BoNAC019*-OE plants than that in WT plants under drought conditions (Fig. 5F–H). These results were consistent with the antioxidant enzyme activities, indicating that overexpressing *BoNAC019* accumulated more ROS by reducing antioxidant enzyme activities to scavenge ROS.

### Overexpression of BoNAC019 reduces anthocyanin accumulation by decreasing expressions of anthocyanin genes

**Figure 6:**
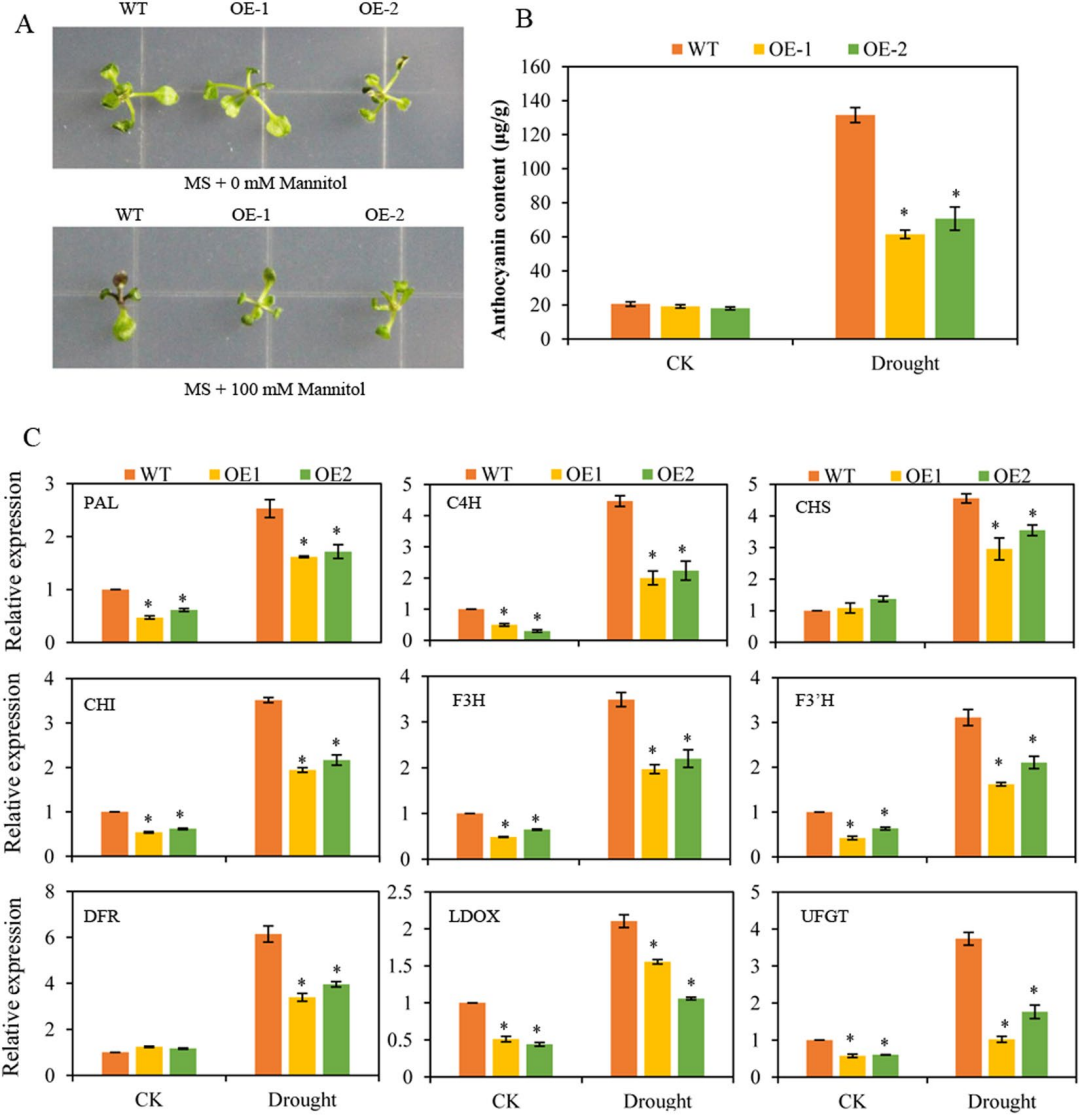
Regulation of dehydration-induced anthocyanin in WT and OE plants. (**A**) Phenotype of 5 days old WT and OE seedlings on MS medium containing 0 and 100 mM mannitol for 5 days. (**B**) Anthocyanin content of WT and OE plants under normal and drought condition. (**C**) The expression of anthocyanin biosynthesis genes in WT and OE plants under normal and drought conditions were analyzed by qPCR. Seedlings on MS medium containing 0 mannitol were used as a mock control (CK). Error bars show the standard deviations for three independent replicates. Asterisks indicate statistically significant differences compared to WT at the same time point (*P < 0.05).

Anthocyanin is a flavonoid that plays important roles in plants. Previous studies have shown that accumulating flavonoids enhances abiotic stress tolerance by improving ROS scavenging ability^35,60,61^. As shown in Fig. 6A, with 100 μM mannitol treatment, the leaves of WT plants exhibited a deeper purple than that of OE plants, and anthocyanin content increased six-fold in WT plants and two-fold in *BoNAC019*-OE plants (Fig. 6B). Under normal conditions, there is almost no anthocyanin accumulation.

In addition, to test whether *BoNAC019* participated in anthocyanin biosynthesis, we detected the expressions of the anthocyanin biosynthetic genes (*PAL*, *C4H*, *CHS*, *CHI*, *F3H*, *F3′H*, *DFR*, *LDOX*, and *UFGT*) in WT plants and *BoNAC019*-OE plants under normal and drought conditions. Expressions of these anthocyanin biosynthetic genes (*PAL*, *C4H*, *CHS*, *F3H*, *ANS*, and *UFGT*) were lower in *BoNAC019*-OE plants than that in WT plants under normal and drought conditions, which was consistent with the anthocyanin content results (Fig. 6C).

The MYB (*TT2*, *PAP1*, *PAP2*, *MYB113*, and *MYB114*) and bHLH (*TT8*, *GL3*, and *EGL3*) transcription factors interacted with WD40 protein (*TTG1*) to regulate the anthocyanin biosynthetic genes. Therefore, we detected the expression levels of these transcription factors. Under normal and drought conditions, only the expressions of TT2, MYB113, and TT8 were much lower in *BoNAC019*-OE plants than that in WT plants. No obvious differences were observed between the expressions of other genes in *BoNAC019*-OE plants and WT plants (Fig. S4). These results indicated that overexpressing of *BoNAC019* reduced anthocyanin accumulation by decreasing the expressions of anthocyanin biosynthetic genes.

### Stress-responsive genes are regulated in BoNAC019 overexpressing plants under drought stress

**Figure 7:**
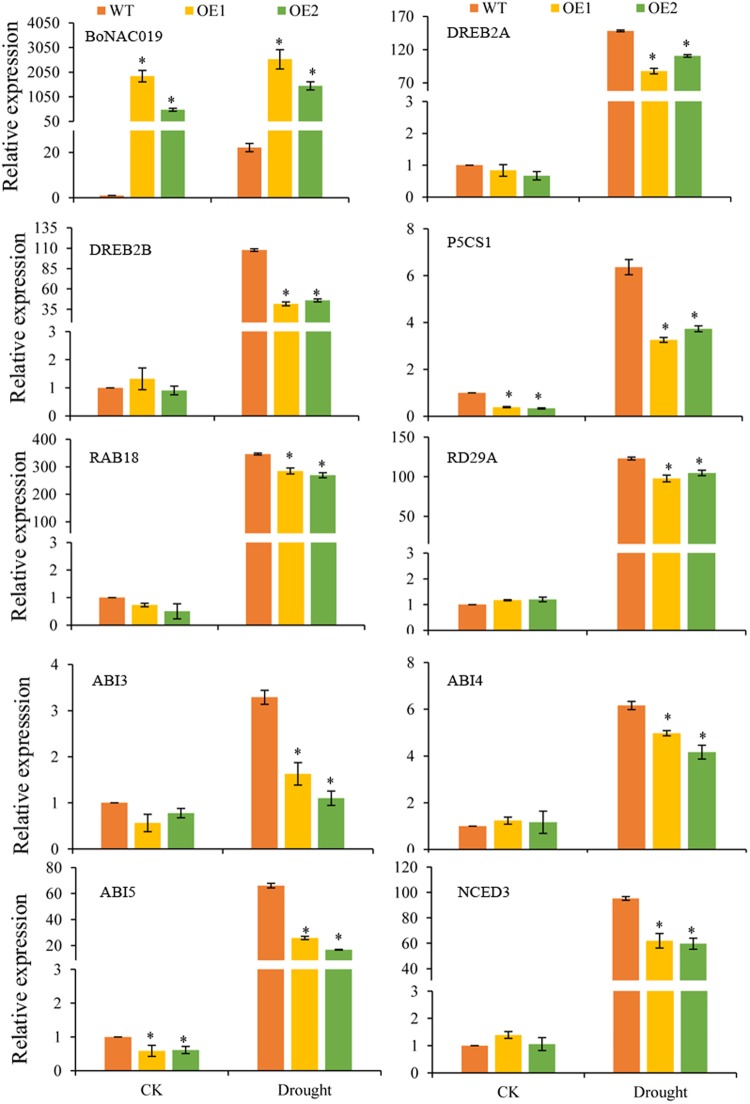
Expression levels of stress responsive genes of WT and OE plants. Cabbage seedlings treated without water were used as a mock control (CK). Error bars show the standard deviations for three independent replicates. Asterisks indicate statistically significant differences compared to WT at the same time point (*P < 0.05).

We selected several stress-responsive genes and compared their expressions in *BoNAC019*-OE plants and WT plants to further investigate the molecular mechanism of *BoNAC019*. Overexpression of *BoNAC019* reduced drought tolerance, therefore, eight stress-related genes (*AtDREB2A, AtDREB2B, AtRD29A, AtRAB18*, and *AtP5CS1*) were selected for further study. Under normal conditions, the expressions of *AtP5CS1* and *AtRAB18* were significantly lower in *BoNAC019*-OE plants. All the stress responsive genes were induced, and the expressions were much lower in BoNAC019-OE plants (Fig. 7).

**Figure 8:**
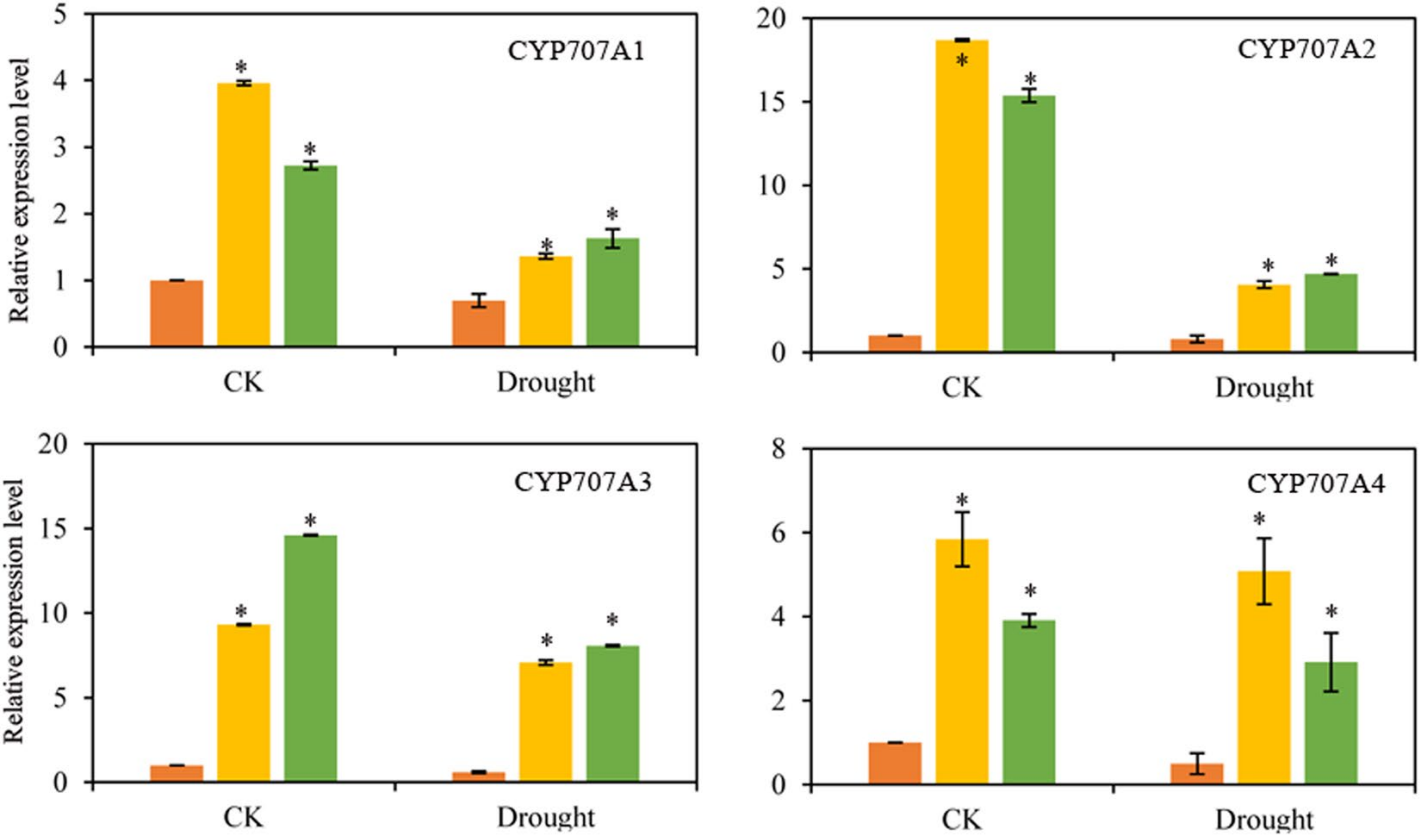
Expression levels of ABA catabolism genes of WT and OE plants. Cabbage seedlings treated without water were used as a mock control (CK). Error bars show the standard deviations for three independent replicates. Asterisks indicate statistically significant differences compared to WT at the same time point (*P < 0.05).

ABA synthetic gene (*AtNCED3*), ABA catabolism genes, ABA signaling genes (*AtPYL1, AtPYL3, AtPP2CA, At*SnRK2.2, *AtSnRK2.4*, and *AtSnRK2.6*) and ABA response genes (*AtABI3, AtABI4*, and *AtABI5*) were selected for further study. Under normal condition, the expressions of ABA catabolism genes (*AtCYP707A1*- *AtCYP707A4*) were significantly higher and the expressions of ABA response genes *AtABI3* and *AtABI5* were much lower in *BoNAC019*-OE plants. No obvious difference of the ABA synthetic gene (*AtNCED3*) and ABA signaling genes expressions were detected between *BoNAC019* OE plants and WT plants. In *BoNAC019*-OE plants, these stress responsive genes expressions were much lower compared to WT plants under drought condition (Fig. 8 and S5).

## Discussion

### Unlike positive regulation of AtNAC019, overexpression of BoNAC019 reduces drought tolerance and decreases expressions of stress responsive genes

Overexpression of *BoNAC019* reduced drought tolerance in *Arabidopsis*, as *BoNAC019*-OE plants showed a lower survival rate and lower RWC, indicating that overexpression of *BoNAC019* reduced drought tolerance by lowering the plants water retaining abilities. Proline accumulation acts in stress-related signaling and cross tolerance^62–64^. The P5CS gene encodes elta-1-pyrroline-5-carboxylate synthase (P5CS) and is an important stress responsive gene in plants^65–67^. Overexpressing *AtP5CS1* increased drought tolerance in transgenic plants^48,68–71^. In this study, the expression of *AtP5CS1* was lower in *BoNAC019*-OE plants than that in WT plants under normal and drought conditions. These results were consistent with the proline content of *BoNAC019*-OE plants. *AtP5CS1* might be the target gene of *BoNAC019*. Further study is needed to verify the target gene of *BoNAC019*.

DREB belongs to the AP2 (APETALA2)/ERF superfamily and participates in the abiotic stress response. DREB1 and DREB2 are two members of DREB transcription factors. DREB1 respond to low temperatures, and DREB2 respond to drought and salt treatments^72–75^. *AtRD29A* and *AtRAB18* were stress and ABA responsive marker genes, and their overexpression enhanced drought tolerance and reduced water loss rate under drought conditions^46,76,77^. *AtDREB2A, AtDREB2B, AtRD29A*, and *AtRAB18* were selected for further study, as expressions of these genes were induced by dehydration, while the expressions of these stress genes were down-regulated in *BoNAC019* OE plants (Fig. 7), indicating that overexpression of *BoNAC019* reduced drought tolerance and increased water loss by downregulating stress responsive genes. *BoNAC019* may regulate other stress responsive genes or interact with other factors to participate in a complex drought signaling pathway; however, further study is needed to confirm this hypothesis.

*AtNAC019* was a well-known stress-responsive NAC transcription factor, and overexpression of *AtNAC019* enhanced drought tolerance (Fig. S3) and upregulated stress gene *AtERD1*^16^. Although *BoNAC019* is highly homologous with AtNAC019, the function of *BoNAC019* in drought stress was completely different. The N-terminal regions which were the DNA binding domains (1–150aa) of BoNAC019 and AtNAC019 were basically the same. The function of the NAC transcription factors was determined by the C-terminal region, and the alignment analysis showed that the C-terminal region of BoNAC019 had a low similarity to AtNAC019 (Fig. S6), which might result in different regulation of downstream genes and different functions in drought stress responses.

### BoNAC019 mediates drought tolerance via an ABA-dependent pathway

ABA is involved in abiotic stress resistance in plants^57,58^ and meditates a series of developmental processes, including seed germination, root elongation, and stomatal movement^78,79^. Abscisic acid is a key endogenous messenger in plants, and it has a crucial role in various plant stresses^80–82^. After drought treatment, the ABA content was induced and much lower in OE plants (Fig. 3E). 9-cis-epoxy carotenoid dioxygenase (NCED) the key enzyme in ABA biosynthesis. *AtNCED3* was induced strongly by dehydration, and overexpression of *AtNCED3* promoted synthesis of ABA. Endogenous ABA content increases to participate in a series of physiological and cellular processes to resist water deficit stress^83,84^. The CYP707A family contains four members in *Arabidopsis*. ABA 80-hydroxylase was coded by CYP707A1- CYP707A4. This enzyme plays a key role in ABA catabolism and in mediating plant response to adverse stress conditions. Suppressing the expressions of CYP707As in cherry fruit by RNAi enhanced drought tolerance in cherry fruits, the water loss rates of transgenic plants were much lower than WT plants^85^. Our results showed that under normal condition, no obvious difference of AtNCED3 expression was detected in *BoNAC019*-OE and WT plants. Under normal and drought conditions, the expressions of CYP707A1-CYP707A4 were induced in *BoNAC019*-OE plants (Fig. 7 and 8).

The signaling mechanism of ABA is critical for improving plant tolerance to stress environments. ABA signaling contains three core components: pyrabactin resistance (PYR)/pyrabactin resistance-like (PYL)/regulatory component of ABA receptors (RCAR), protein phosphatase 2C and SNF1(Sucrose non-fermenting)- related protein kinase 2. In the presence of ABA, PP2C activity which functioned as a negative regulator was inhibited by PYR/PYL/RCAR-PP2C complex formation. Inhibition of PP2C activity activated SnRK2 and then phosphorylates downstream substrate proteins such as transcription factors, and thus facilitating transcription of ABA-responsive genes^81^. The ABI3, ABI4, and ABI5 proteins have been reported to participate in seed germination and early seedling growth and development^86–88^. ABI5 was induced by dehydration treatment, and overexpression of ABI5 enhanced stress tolerance^86,89^. In addition, the expression levels of *AtNCED3*, ABA signaling genes, ABA response genes were lower in *BoNAC019*-OE plants than those in WT plants, while no obvious difference of these genes expressions were detected under normal conditions (Fig. 7 and S5). BoNAC019 might activate the ABA catabolism genes to decrease the tolerance to drought stress. These genes might be the target genes of BoNAC019. The other genes involved in ABA biosynthesis, ABA signaling pathway and ABA response genes were not directly regulated by BoNAC019. BoNAC019 might interact with the other factors to regulate these genes expressions. BoNAC019 might negatively regulate dehydration response by inducing ABA catabolism genes, resulting in decreasing ABA content and drought tolerance.

### Overexpression of BoNAC019 reduces ROS scavenging ability by decreasing antioxidant enzyme activities and anthocyanin accumulation

Enzymatic antioxidants include SOD, POD, and CAT. Overexpression of these genes encoding relevant enzymes improved ROS scavenging ability to protect plants against abiotic stress^90–92^. Under drought conditions, overexpressing plants accumulated more H_2_O_2_, and the activities of the antioxidant enzymes were lower than those in WT plants (Fig. 5A–E). Expressions of *AtSOD*, *AtPOD*, and *AtCAT* encoding antioxidant enzymes were detected in *BoNAC019*-OE plants and WT plants, and their expression levels all decreased in *BoNAC019*-OE plants under the drought condition (Fig. 5F–H). Therefore, *BoNAC019* probably participates in ROS scavenging by reducing antioxidant enzyme activities.

Non-enzymatic antioxidants include ascorbate, GSH, carotenoids, tocopherols, and flavonoids^31^. Anthocyanin is flavonoid that plays important roles in plants. Previous studies have shown that flavonoid accumulation could enhance abiotic stress tolerance by improving ROS scavenging ability^35,60,61,93^. In this study, the anthocyanin content of *BoNAC019*-OE plants was significantly lower than that of WT plants under drought conditions (Fig. 6A).

Synthesis of anthocyanins in *Arabidopsis* is well understood^40,94,95^. *PAL*, *C4H*, *CHS*, *CHI*, *F3H*, *F3′H*, *DFR*, *LDOX*, and *UFGT* are important enzymes in the anthocyanin biosynthetic pathway^96,97^. In this study, the expression levels of these genes were lower in *BoNAC019*-OE plants than those in WT plants under normal and drought conditions, indicating that *BoNAC019* participates in anthocyanin biosynthesis by regulating the anthocyanin biosynthetic genes (Fig. 6B).

MYB, bHLH, and WD40 are three important transcription factors involved in anthocyanin biosynthesis and directly regulate the anthocyanin biosynthetic genes^40,98,99^. MYB12 regulates the expressions of *CHS, CHI*, and *F3H*^100^. Expressions of *DFR* and *ANS* were dramatically induced by overexpressing *FtWD40*^101^. PtrMYB75 interacted with bHLH113 and TTG1 to repress the expressions of anthocyanin biosynthetic genes by directly binding to the promoters of these genes in poplar^102^. In this study, we also detected the expressions of these above transcription factors. Under normal and drought conditions, only TT2 and MYB113 expression levels were significantly lower in *BoNAC019*-OE plants than those in WT plants. No obvious difference was observed between the expressions of other genes in *BoNAC019*-OE plants and WT plants (Fig. S4). Therefore, we hypothesized that WD40 and bHLH might be the upstream transcription factors of *BoNAC019* or that WD40 and bHLH might interact with BoNAC019 to regulate the anthocyanin biosynthetic genes. Li *et al*. reported that suppressing CPY707A2 by RNAi in cherry fruit induced anthocyanin accumulation and upregulating the anthocyanin biosynthesis genes^85^. In this study, we found that the expressions of CPY707A genes were activated by BoNAC019 (Fig. 8) and the ABA content (Fig. 3E) was significantly lower in OE plants. Therefore, we speculated that BoNAC019 might inhibit the synthesis of anthocyanin by activating the expression of CPY707A genes. Further study is needed to confirm this hypothesis.

In summary, overexpressing BoNAC019 showed decreased drought tolerance and accumulated more ROS by decreasing antioxidant enzyme activities and anthocyanin accumulation to scavenge ROS, the ability to scavenge ROS was weaker in *BoNAC019*-OE plants than that in WT plants. Relevant studies of NAC transcription factors participating in anthocyanin biosynthesis in response to drought stress are limited. Our study provides some new insight into the molecular mechanism of drought resistance in the Brassica family.

## Electronic supplementary material

supplementary information
