# Supplementary material for: Overexpression of BoNAC019, a NAC transcription factor from Brassica oleracea, negatively regulates the dehydration response and anthocyanin biosynthesis in Arabidopsis
Source: Sci Rep. 2018 Sep 6;8:13349. doi: 10.1038/s41598-018-31690-1 (PMC6127341; doi:10.1038/s41598-018-31690-1)
Supplement: Supplementary file 1 — supplementary information [file 41598_2018_31690_MOESM1_ESM.pdf]

Overexpression of *BoNAC019*, a NAC transcription factor from *Brassica oleracea*, negatively regulates the dehydration response and anthocyanin biosynthesis in *Arabidopsis*

Jinfang Wang[1#], Weiran Lian[1#], Yunyun Cao[1#], Xiaoyun Wang[1], Gongle Wang[1], Chuandong Qi[1], Lun Liu[1], Sijia Qin[1], Xiaowei Yuan[2], Xingsheng Li[2], Shuxin Ren[3] and Yang-Dong Guo[1*]

[1] *College of Horticulture, Beijing Key Laboratory of Growth and Developmental Regulation for Protected Vegetable Crops, China Agricultural University, Beijing, 100193, China*

[2] *Shandong Huasheng Agriculture Co., Ltd, Shandong, China,*

[3] *School of Agriculture, Virginia State University, PO Box 9061, Petersburg, VA 23806, USA*

Corresponding author: Yang-Dong Guo, College of Horticulture,

China Agricultural University, 2#, Yuanmingyuan West Rd, Beijing 100193, China.

Tel: (86-10)-62734845;

Fax: (86-10)-62733404;

E-mail: yaguo@cau.edu.cn

Table S1. List of primers used in this study.

Purpose	Gene	Primer
ORF of NAC gene	*BoNAC019*<br>Bol039157	F: ATGGGTATCCAAGAAACCGA<br>R: TCATATATAACCAAACCCACCA
QPCR of CsNAC gene	*BoNAC019*<br><br>*BoActin*	F: GGTTCAACGGTTTAGCGGACA<br>R: CCCACCAACTTGCCCTGAATA<br>F: TAACAGGGAGAAGATGACTCAGATCA<br>R: AAGATCAAGACGAAGGATAGCATGAG
OE construct	*BoNAC019*	F: ggactctagaggatccccggg ATGGGTATCCAAGAAACCGA<br>R: ataagggactgaccacccggg TCATATATAACCAAACCCACCA
Subcellular location construct	*BoNAC019*	F: accatggtagatctgactagt ATGGGTATCCAAGAAACCGA<br>R: aagttcttctcctttactagtTATATAACCAAACCCACCA
Transactivation assay	*BoNAC019*<br><br>*BoNAC019*-N<br><br>*BoNAC019*-C	F: GGAATTCC ATGGGTATCCAAGAAACCGA<br>R: GAAGATCTTC TCATATATAACCAAACCCACCA<br>F: GGAATTCC ATGGGTATCCAAGAAAC<br>R: GAAGATCTTC CCGTTTGCACGAGAGGG<br>F: GGAATTCC AAGCTCTAAGTTAGATG<br>R: GAAGATCTTC TCATATATAACCAAACCC
QPCR of stress-responsive genes	*AtRD29A*<br>*AT5G52310*<br>*AtRAB18*<br>*AT5G66400*<br>*AtDREB2A*<br>*AT5G05410.*<br>*AtDREB2B*<br>*AT3G11020*<br>*AtP5CS1*<br>*AT2G39800*<br>*AtABI3*<br>*AT3G24650*<br>*AtABI4*<br>*AT2G40220*<br>*AtABI5*<br>*AT2G36270*<br>*AtNCED3*<br>*AT3G14440*<br>*AtSOD*<br>*AT1G08830*<br>*AtPOD*<br>*AT1G14540*<br>*AtCAT*<br>*AT1G20630*	F: TCTTGATGGTCAACGGAAGGTC<br>R: CCGGTACTCCTCCAGTTTCTTC<br>F: GGGAGGAGGAAGAAGGGAATA<br>R: CGTAGCCACCAGCATCATATC<br>F: ATTGTCTGGAGAATGGTGCGG<br>R: GATAGCGAATCCTGCTGTTGT<br>F: AACCGAAGCAGGAAGAAGAGG<br>R: TTTTGGTCTTGGCTCTGATGG<br>F: TGTGTGTTTGTGTATTTGGTTGAGAC<br>R: TGAGTACTAAGCAGAGAGGAAACAAAA<br>F: TGGAGACGCTGATGATACTGT<br>R: AGGAGACGATTGCGTTGACAG<br>F: GGCGTTAGGGCAGGAACAAGG<br>R: AATCCCAAATACTCCCCCAAT<br>F: AAGAGAGGGATAGCGAACGAG<br>R: CTAACCACACCAGCCTTCACC<br>F: CAATCATCAAACTCTCCCGCC<br>R: TCTCGTGGCTGACAAGGAAAC<br>F: ATCTCCTCCCTGCTTTCAACAAC<br>R: CCCAGACATCAATGGTAAGCAAT<br>F: CGGCAAGAGGATTTGAAGTTA<br>R: TGTCTGCGATAGCCCTAAACG<br>F: TCACTTGTGCTGACTTTCTCCGA<br>R: TCTTGGATGTGAGATTTTGGGTT

	AtPAL1	F: GAGGAGTGGACGCTATGTTATGC
	AT2G37040	R: CACCATAACTATCAGTGCCTTTG
	AtCHS	F: CTTCCCTCAAATGTCCGTCTATG
	AT5G13930	R: CGTGTCCTCGTTGTCTGCTCT
	AtCHI	F: CCTTTTCGTCCTTGTTCTTCATCAT
	AT3G55120	R: GAGGCGGTTCTGGAATCTATCA
	AtC4H	F: ATCTCAACCACCGTAATCTCGTC
	AT2G30490	R: TTCCTCCAATGCTCGCCGTAAAC
	AtF3H	F: TGGGTGAAAGTGACGGAGGAGTA
	AT3G51240	R: GTAATGGTTCCAGGGTCAGTGTG
	AtF3'H	F: GACACCGATGGAGACTGTTGAGA
	AT5G07990	R: AGCGACGCCTTGTAAATCTAACC
	AtDFR	F: AGGCGGCATAGACGTTGTGA
	AT5G42800	R: GGAGAAAGCAGCGTGGGATT
	AtANS	F: CTTTTCTACGAGGGCAAATGGGT
	AT4G22880	R: CACAGCCCAAGAAATCCTAACCT
	AtUF3GT	F: TCCACACCATCTCTATCCCTCAG
	AT5G54060	R: AGAAGGGACAAGAGACAGTGCGA
	AtTT2	F: GATGGATCAAGGTGGATCTTCG
	AT5G35550	R: AAGTGAAGTCTCGGAGCCAATC
	AtMYB113	F: ATCTTGTTCTTCGCCTTCATAAA
	AT1G66370	R: TCGTTCATCGTGCTTCTTACTCA
	AtMYB114	F: AAGTATGGAGAAGGCAAATGGCA
	AT1G66380	R: GTAGTTCTTGACATCATTAGCGG
	AtPAP1	F: CCATCTCAATGCCCCACCA
	AT1G56650	R: TTCTGTTGTCGTCGCTTCAGG
	AtPAP2	F: ATAGGTGGTCCTTGATTGCTGG
	AT1G66390	R: AGGATCGAGGTCGAGGCTTAA
	AtTTG1	F: TTGGAACGATGTAGAGCCGAAAC
	AT5G24520	R: CAGGCTGAGGACTCTCGTAAATG
	AtTT8	F: GCAACAGCATAAGCGGACG
	AT4G09820	R: CCTCTTACTTTCGCCCTTATCTCC
	AtEGL3	F: TTCAAGCGATTGCGTTTCCC
	AT1G63650	R: GATGATCGCTTCCACCTTGTGA
	AtGL3	F: TTTGCTTCCCGTTCCTTGG
	AT5G41315	R: AGGCTCCGTACTGAACATAGGC
	AtACTIN	F: CTGGAATGGTGAAGGCTGGTT
	AT1G13320	R: CGATTGGATACTTCAGAGTGAGGAT
	CYP707A1	F: AGAGTCTCTAACTTGGGGAGAT
	AT4G19230	R: TGGATCAAATTTCCCCGGATTA
	CYP707A2	F: ATTTAAATGGTGGTTGCACTGG
	AT2G29090	R: GCGAAGAAGGAATTGGGATTTT
	CYP707A3	F: TGATATATTTTCGGATCCGGGG
	AT5G45340	R: TGATATATTTTCGGATCCGGGG

	CYP707A4<br>*AT3G19270*<br>*AtPP2CA*<br>*AT3G11410*<br>*PYL1*<br>*AT5G46790*<br>*PYL6*<br>*AT2G40330*<br>*SnRK2.2*<br>*AT3G50500*<br>*SnRK2.4*<br>*AT1G10940*<br>*SnRK2.6*<br>*AT4G33950*	F: TTTAGCTAACTCATTGCCTGGA<br>R: TGCCACTGACACATAAGGTTAT<br>F: GTTAATGGTGCTACTCGGAGTA<br>R: GTGATCTACGGAGAGAGGAATG<br>F: GTGAGTGAAGATTTCGAGATGC<br>R: CGATCATCGTCCAACAGATCTA<br>F: ACAAACACTTCGTGAAAAGCTG<br>R: TGTAGTTCATGAGTCTGTGGTC<br>F: TTAAAGAGGTGATTTTGACGCC<br>R: AATCCGCTCATAAAGTTCTCCA<br>F: AAGGGAACAAGAGAAAACATGC<br>R: GAGCTTCAGATCTCTATGGCAT<br>F: ATATCTCCTGAATGTCGCCATT<br>R: GTTCATTAGATCTGCCGGTAGA

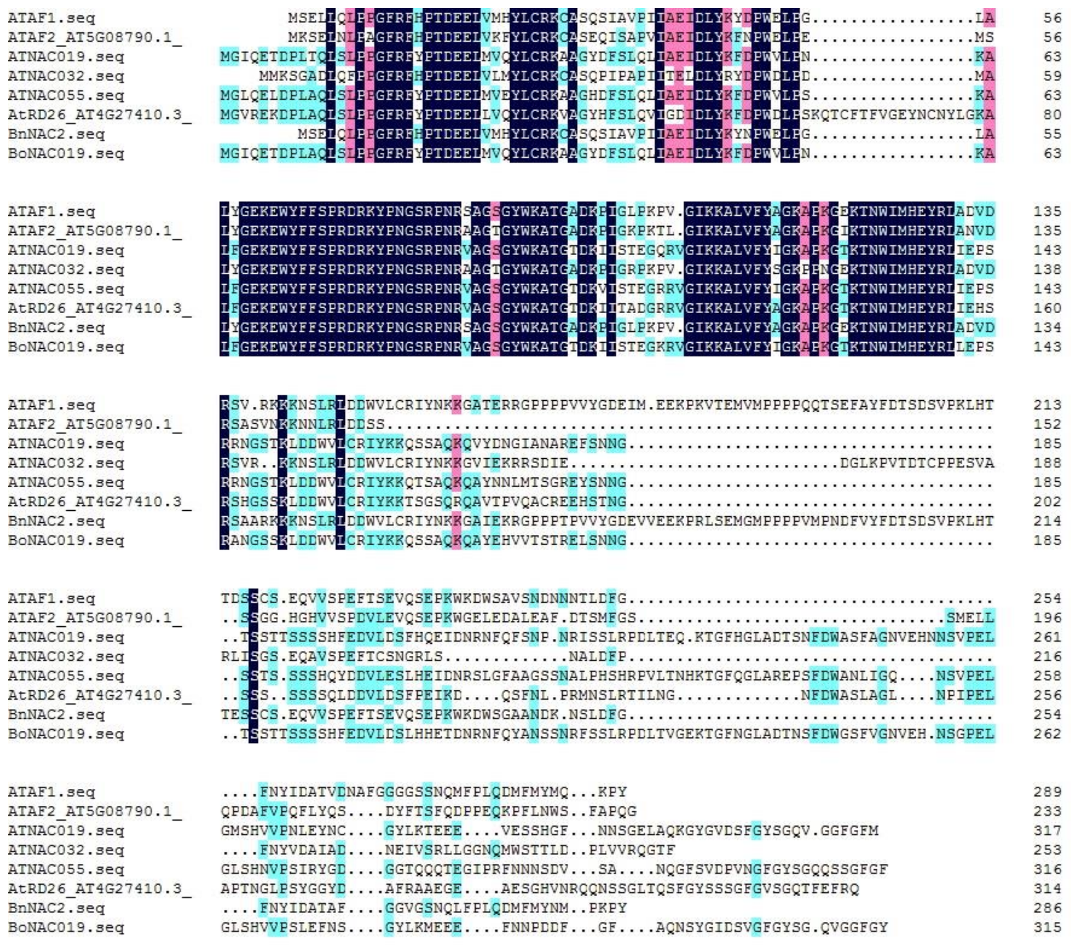

Fig. S1. Sequence analysis of BoNAC019. Amino acid alignment of BoNAC019 and other NAC family members from selected plant species, including AtNAC032 (GenBank accession no. NP_177869.1), ATAF1 (GenBank accession NP_171677.1), ATAF2 (GenBank accession: X74756.1), AtNA019 (GenBank accession: NM_104167.6), AtNAC055 (GenBank accession: NM_112418.4), AtRD26 (GenBank accession: NM_118875.4) from *Arabidopsis*, BnNAC2 (GenBank accession no. NP_001302866.1) from *Brassica napus*. The amino acid residues are exactly the same as the black shadow, the 75% is the same as the pink shadow, and the 50% is the same as the blue shadow Alignments were performed using DNAman.

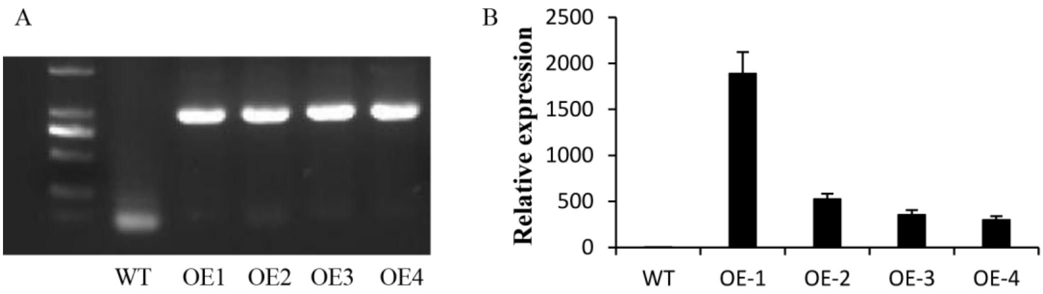

Fig. S2. PCR and qPCR analysis of transgenic Arabidopsis plants. A. PCR analysis of overexpressing transgenic Arabidopsis plants. B. qPCR analysis of overexpressing transgenic Arabidopsis plants. Error bars show the standard deviations for three independent replicates.

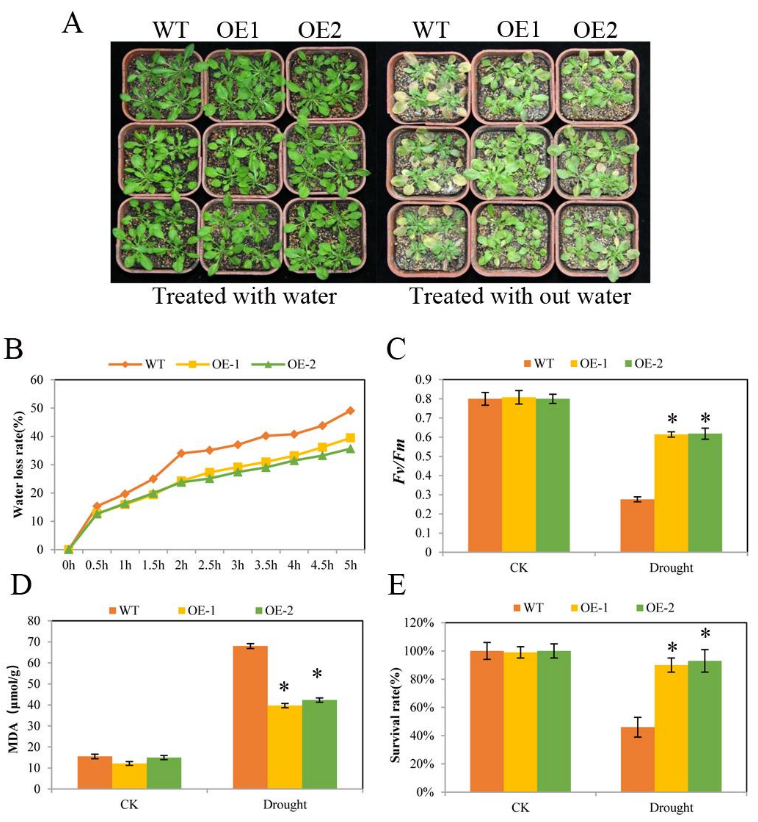

Fig. S3. Overexpression of AtNAC019 enhanced tolerance to drought stress. A. Phenotypes of WT and OE plants after water withholding for three weeks. B. Water loss rate. C. *Fv/Fm* ratios. D. MDA content. E. Survival rate of WT and OE plants after drought treatment. Seedlings treated with water were used as a mock control (CK). Error bars show the standard deviations for three independent replicates. Asterisks indicate statistically significant differences compared to WT at the same time point (*$P < 0.05$).

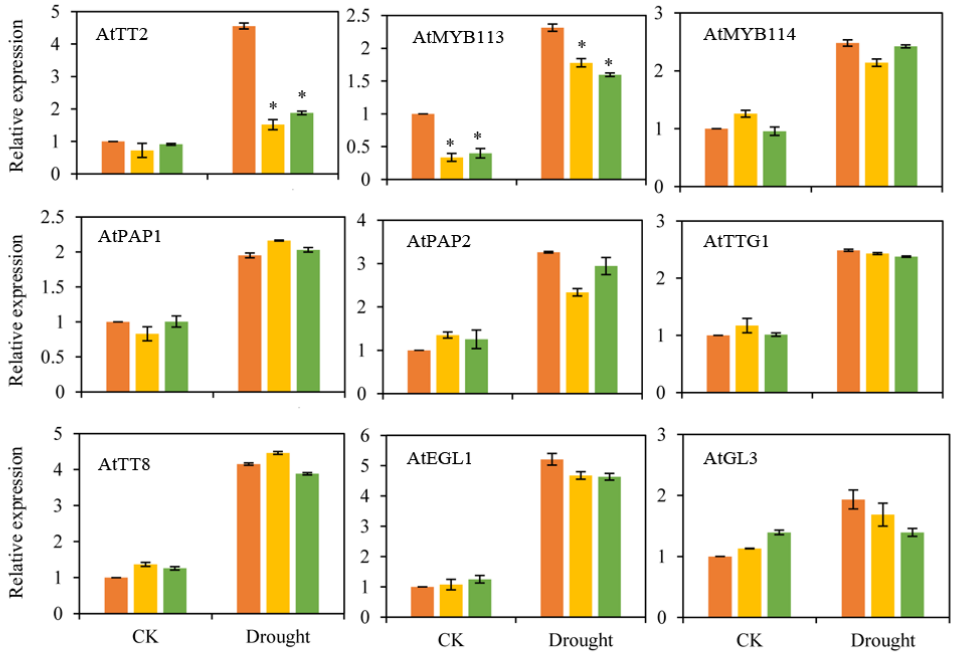

Fig. S4. Expression levels of transcription factors involved in anthocyanin biosynthesis of WT and OE plants. Cabbage seedlings on MS medium containing 0 mannitol were used as a mock control (CK). Error bars show the standard deviations for three independent replicates. Asterisks indicate statistically significant differences compared to WT at the same time point (*P < 0.05).

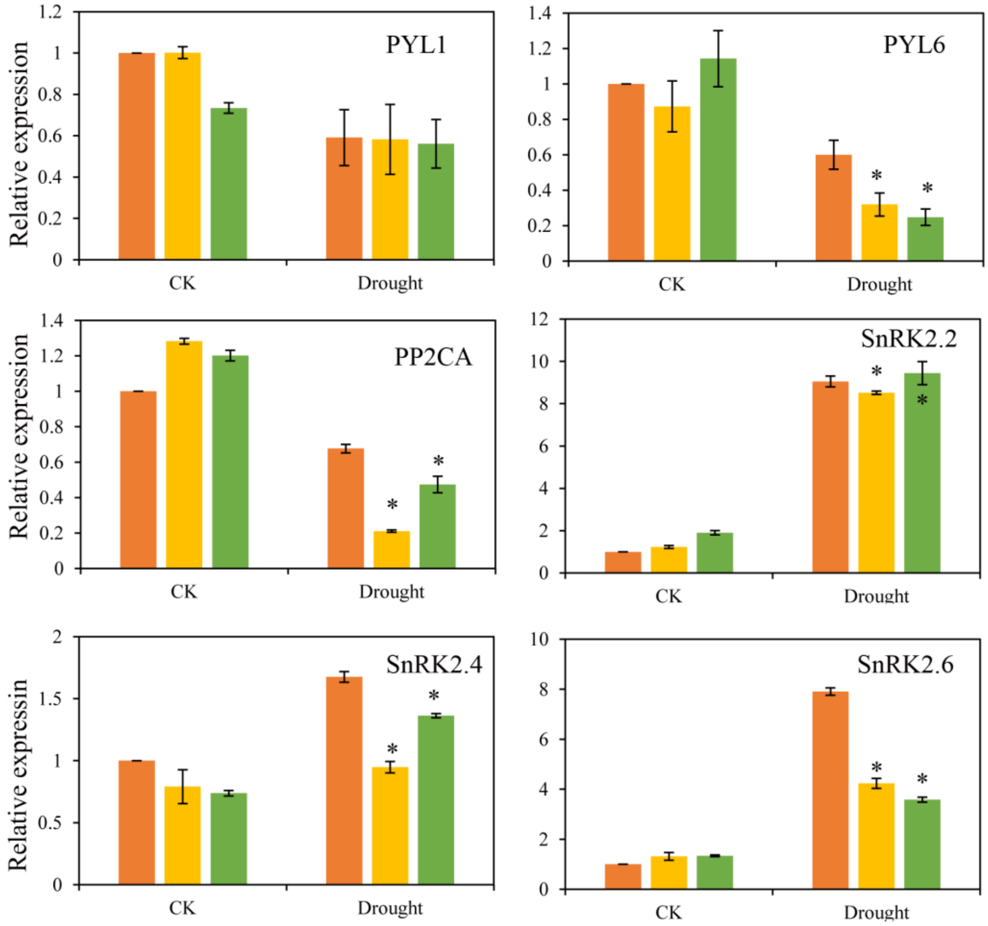

Fig. S5. Expression levels of ABA signaling genes of WT and OE plants. Cabbage seedlings treated without water were used as a mock control (CK). Error bars show the standard deviations for three independent replicates. Asterisks indicate statistically significant differences compared to WT at the same time point (*$P < 0.05$).

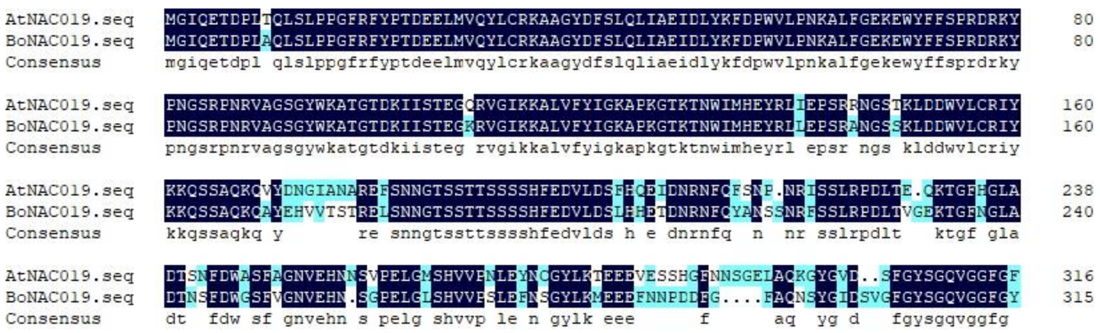

Fig. S6. Sequence analysis of BoNAC019 and AtNAC019. The amino acid residues are exactly the same as the black shadow and the 50% is the same as the blue shadow Alignments were performed using DNAman.
